# Ostarine and Ligandrol Improve Muscle Tissue in an Ovariectomized Rat Model

**DOI:** 10.3389/fendo.2020.556581

**Published:** 2020-09-17

**Authors:** Paul Jonathan Roch, Danny Henkies, Jan Christoph Carstens, Carsten Krischek, Wolfgang Lehmann, Marina Komrakova, Stephan Sehmisch

**Affiliations:** ^1^Department of Trauma Surgery, Orthopaedics and Plastic Surgery, University of Göttingen, Göttingen, Germany; ^2^University of Veterinary Medicine Hannover, Foundation, Hannover, Germany

**Keywords:** ovariectomized rats, postmenopausal osteoporosis, selective androgen receptor modulators (SARMs), ostarine, ligandrol, muscle correspondence

## Abstract

In postmenopausal women, hormonal decline changes muscle function and structure. The non-steroidal selective androgen receptor modulators (SARMs) Ostarine (OS) and Ligandrol (LG) have been shown to increase muscle mass and physical function while showing a relative low risk profile. Information about their effects on muscle structure and metabolism is lacking. To analyze this, two experiments were performed using ovariectomized rats as a standard model for postmenopausal conditions. In each experiment, 3-month old Sprague-Dawley rats were divided into five groups (*n* = 12 to 15). One group remained intact (Non-OVX), the other four groups were ovariectomized (OVX) and remained untreated for eight (OS Experiment) or nine (LG Experiment) weeks. Thereafter, rats of three of the four OVX groups were treated with OS or LG (with doses of 0.04, 0.4, or 4 mg/kg body weight/day) for 5 weeks. Then, uterus, gastrocnemius, and soleus muscles were weighed, fiber size, capillary density, and enzyme activity (lactate dehydrogenase [LDH], citrate synthase [CS], and complex I) were analyzed. In the LG experiment, intramuscular fat content was determined in the quadriceps femoris muscle. All OS treatments resulted in a higher capillary density in the gastrocnemius and longissimus muscles compared with the Non-OVX and the OVX rats, whereas all LG treatments showed a higher capillary density compared with the Non-OVX group. Muscle fiber size and distribution patterns were not changed under either SARM. The CS activity was higher in the longissimus muscle under OS treatment. LG resulted in a higher activity of CS in the gastrocnemius and of LDH in the longissimus muscle. Both SARMs showed an uterotrophic effect, OS at 4 and 0,4 mg dosages, LG at 4 mg dosage. In sum, beneficial effect on muscle vascularization was observed for both SARMs with a stronger impact for OS. LG showed more effect on muscle metabolism. However, a higher muscle weight and intramuscular fat content observed after LG treatment (4 mg) as well as an uterotrophic effect of both SARMs at higher dosages could be considered as an unfavorable side effects and might be a limitation for their application at these dosages.

## Introduction

Musculoskeletal disorders account for approximately one-third of diseases and illness of the elderly in industrial nations and continue to show an upward trend (1). Consequently, the risk of falls has increased, and fractures are becoming a more common injury. Instability and injuries after falls are interdependent (2, 3). The primary causes for musculoskeletal disorders are sarcopenia and osteoporosis (1, 4). Both, sarcopenia and osteoporosis are under continuous research and definitions are in change. While osteoporosis may be described with reduced bone mass that results in decreased bone stability and increased fracture risk (5), sarcopenia is considered when low muscle strength, quality or quantity and low physical performance occur (6). In women, hormonal changes such as the decline in estrogen in the postmenopausal metabolism may support the development of both sarcopenia and osteoporosis (5, 7).

Hormone replacement therapy in postmenopausal women is associated with severe side effects, such as an increased risk of coronary heart disease, breast cancer, stroke, and venous thromboembolism (8, 9). Nevertheless, it is still the most effective treatment for postmenopausal symptoms and for carefully selected women, benefits could exceed risks (10, 11). Selective estrogen receptor modulators have beneficial effects on the musculoskeletal system like estrogen, they have fewer adverse events on breasts and the uterus. However, they are still associated with venous thromboembolism and stroke (12, 13).

Alternatively, testosterone supplementation has been shown to increase muscle strength in men and to prevent muscle atrophy in orchidectomized male mice (14–17). Although beneficial effects on sexual function, personal distress, and blood lipids were observed in postmenopausal women, administration of testosterone in women is controversial since long-term studies are lacking to prove safety (18). In men, testosterone supplementation is associated with an increased risk of respiratory, cardiac, and dermatologic side effects (19).

Selective androgen receptor modulators (SARMs) were developed to overcome the side effects and the poor oral bioavailability and pharmacokinetic profile of testosterone (20, 21). While the exact mechanism of the action of SARMs is not fully understood, the key reason for higher tissue specificity and more favorable pharmacokinetics is considered to be their resistance to aromatization or 5-α-reduction (22, 23). Ostarine (OS) (enobosarm, S-22, MK-2866, or GTx-024) and Ligandrol (LG) (LGD-4033, VK5211) are both non-steroidal SARMs. OS was been shown to increase the lean body mass and physical function in elderly men and postmenopausal women as well as reduce muscle wasting in patients with cancer (24–26). For LG, an increase in the lean body mass in young healthy patients has been observed (27).

OS and LG are still being investigated in clinical trials, and neither have been approved as treatments to this point (28, 29). There are no studies reporting *in vivo* effects of OS and LG on postmenopausal muscle structure and metabolism. The aim of the present study was to investigate the effect of OS and LG on the muscle tissue of ovariectomized rats as the standard model for postmenopausal conditions (30, 31). Two independent experiments were conducted. The OS effect on bone tissue and animal model as a part of Experiment I has been recently published (32, 33).

## Materials and Methods

### General Procedures

**Figure 1:**
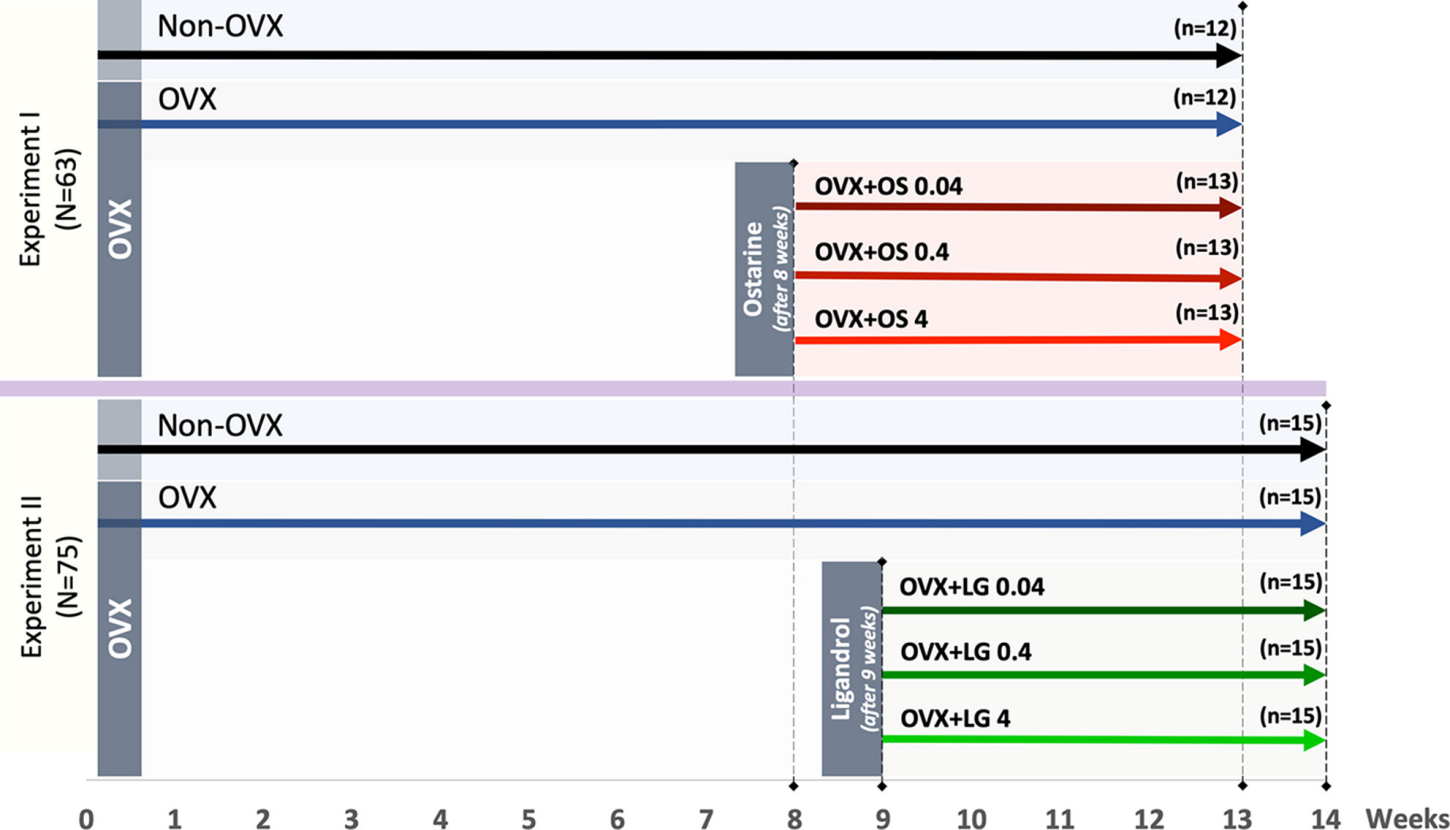
Schematic flowchart of the two experiments: Experiment I: Ostarine (OS) and Experiment II: Ligandrol (LG). Three-month-old female rats were either ovariectomized (OVX) or left intact (Non-OVX). In Experiment I, OVX rats were treated with OS at three different dosages (0.04, 0.4, and 4 mg/kg/BW) after 8 weeks post-OVX, whereas in Experiment II, OVX rats were treated with LG at the same dosages after 9 weeks post-OVX. In both experiments, the treatments were conducted for up to 5 weeks.

The animal study protocol was approved by the local regional government (14/1396, Oldenburg, Germany) in accordance with German animal protection laws prior to performing the study. Three-month old Sprague-Dawley rats (Fa. Janvier Labs, Saint-Berthevin, France) were ovariectomized (OVX) or left intact (Non-OVX). Changes in musculoskeletal system caused by estrogen deficiency are known to develop in rats after at least 4–6 weeks post-OVX (30, 31). The treatments with OS were initiated 8 weeks after OVX and with LG after nine weeks according to the design of the two experiments (Figure 1). Three to four rats were housed in cages (Type Makrolon® IV, Techniplast Deutschland GmbH, Hohenpreißenberg, Deutschland), four cages per treatment group.

In Experiment I, the effect of the SARM OS on the muscle structure and metabolism was studied. Rats were randomly divided into five groups (Figure 1): Group 1: Non-OVX (*n* = 12); Group 2: OVX (*n* = 12); Groups 3 to 5: OVX rats treated with OS at three different dosages of 0.04, 0.4, and 4 mg/kg/body weight (BW) (OVX+OS 0.04 [*n* = 13], OVX+OS 0.4 [*n* = 13]), and OVX+OS 4 [*n* = 13]), respectively). Data on BW, uterine weight, muscle weight, and OS dosages have been previously published (32, 33).

In Experiment II, the effect of SARM LG on the muscle was studied. Rats were divided into five groups (Figure 1): Group 1: Non-OVX (*n* = 15); Group 2 (*n* = 15): OVX; and Groups 3 to 5: OVX rats were treated with LG at three different dosages of 0.04, 0.4, and 4 mg/kg/BW (OVX+LG 0.04 [*n* = 15], OVX+LG 0.4 [*n* = 15]), and OVX+LG 4 [*n* = 15], respectively).

In both experiments, a low dosage of 0.04 mg/kg BW/day was calculated based on a human-equivalent dose sufficient for improvements of total lean body mass and physical function (24). To investigate dose-dependent effects, 10-fold and 100-fold dosages were used. The treatments with SARMs were conducted for up to 5 weeks (Figure 1).

All rats received a soy-free rodent diet (ssniff Spezial Diät GmbH, Soest, Germany) throughout the experiment. OS and LG were supplied with the soy-free diet (ssniff Spezial Diät GmbH). The OS (MK-2866) was obtained from Shanghai Biochempartner Co., Ltd. (Shanghai, China), whereas the LG (LGD-4033) was obtained from Hölzel Diagnostika Handels GmbH (Cologne, Germany). All rats received food and demineralized water *ad libitum*. Food intake and BW were recorded weekly. The remaining food in the cage was weighed weekly to calculate the average daily food intake of a rat by dividing these data by days between the weighing and number of rats in a cage. The average daily dosage of OS and LG was calculated based on the daily food intake and the mean BW in the cage on the respective week (34). After 13 weeks post-OVX, all animals were euthanized under CO_2_ anesthesia. Blood serum was collected for further analysis of creatine kinase (CK) as a marker of muscle damage (35). The uterus was weighed. The gastrocnemius muscle (GM), soleus muscle (SM), and longissimus muscle (LM) were extracted. The GM and SM were weighed and all muscles were frozen in liquid nitrogen (36) and stored at −80°C until further analyses. Either left or right muscles were used randomly in either histological or enzyme analyses. In Experiment II, intramuscular fat content was determined in the quadriceps femoris muscle in Non-OVX, OVX, and OVX+LG 4 groups.

### Histological Analyses

Two staining methods were applied for analyses of muscle capillaries and muscle fibers. Serial cross-sections of a 12-μm thickness were cut from the middle part of each muscle using a cryotome (CM 1900; Leica Microsystems, Wetzler, Germany) at −20°C, air-dried, and stored at −20°C for further stainings. All chemicals were obtained from Merck KGaA (Darmstadt, Germany) unless otherwise indicated.

### Staining of Muscle Capillaries

Staining of muscle capillaries was performed using a modified periodic acid-Schiff (PAS) reaction (37). A fixative solution (ethanol/chloroform/glacial acid in a 16:3:1 ratio) was applied on the serial cross-sections for 1 h at 4°C and subsequently for 10 min at room temperature. Sections were washed 10 times with distilled water. Next, a-Amylase from porcine pancreas (Sigma-Aldrich Laborchemikalien GmbH, Seelze, Deutschland) in 0.3% (w/v) water solution was used for 25 min at 37°C for hydrolyzation of polysaccharides. The sections were then repeatedly washed 10 times with distilled water. Next, the PAS reaction was performed: (1) periodic acid of 1% (w/v) was applied for 30 min, (2) sections were washed 10 times with distilled water, and (3) Schiff's reagent solution (Roth, Karlsruhe, Germany) was applied for several minutes (2–25 min) under visual control to avoid overstaining. Following that, washing was performed in three steps: treatment with 10% (w/v) potassium sulfite/1 N HCl/water (1:1:20) (30 min), running tap water (10 min), and distilled water (3 min) (38, 39).

### Staining of Muscle Fibers

A fixative solution consisting of 1% (v/v) paraformaldehyde solution (pH 6^.^6), 1% (w/v) CaCl_2_, and 6% (w/v) sucrose (40) was applied for 1 min. Sections were washed with distilled water twice for five min each. Afterwards, a modified staining method with adenosine-triphophatase (ATPase) was performed as described by Horák (41): (1) Incubation: diaphorase solution (2.4 mM NADH, 27 mM phosphate buffer [pH 7.4], and 0.4 mM nitro blue tetrazolium chloride), at 37°C, humidified atmosphere, 60 min; (2) Washing (distilled water, 15 min); (3) Pre-incubation: 18 mM CaCl_2_ plus 0.4% (v/v) glacial acetic acid, pH 4.2, 15 min; (4) Washing (100 mM Tris(hydroxymethyl)aminomethane (Tris) plus 18 mM CaCl_2_, pH 7.8, 2 min); (5) ATPase incubation: 0.1 M glycine (pH 9^.^4), 18 mM CaCl_2_, 48.8 mM KCl, 2.8 mM ATP, 37°C, 30 min; (6) Washing (3 × 30 s with 68 mM CaCl_2_); (7) Incubation: 84 mM cobalt chloride, 2 min; (8) Washing (3 × 45 s with distilled water); (9) Incubation: 0.08% (v/v) ammonium sulfide solution, 2 min; and (10) Washing (1 × 10 min with running tap water, 1 × 5 min immersing in distilled water) (38, 39).

### Microscopy and Analysis of Muscles

Capillaries and fibers were analyzed at 10-fold magnification (Eclipse E 600 microscope; Nikon, Tokyo, Japan), digital camera (DS-Fi2 Digital Camera; Nikon Instruments Europe, Amsterdam, Netherlands), and analyzed digitally (NIS-Elements AR 4.0 imaging software; Nikon Instruments Europe). Evaluation of fibers was performed in three randomly chosen fields of 1 mm^2^ in the ATPase stained sections. Ninety slow-twitch oxidative and fast-twitch oxidative (STO and FTO; fiber types I and IIa) and 90 fast-twitch glycolytic (FTG; fiber type IIb) fibers were skirted (42). Since SM consists mainly of STO and FTO fibers, only these fibers were measured (43). Fiber contours were manually defined in digital images. Distribution of fibers was analyzed within a 1 mm^2^ field by calculating the percentage of STO+FTO and FTG fibers in LM. Evaluation of capillaries was performed in two randomly chosen fields of 0.25 mm^2^ in the amylase-PAS stained sections. The ratio of capillaries to fibers was calculated (capillary density) (44).

### Analysis of Muscle Enzyme Activity

For analysis of muscle enzyme activities (lactate dehydrogenase [LDH], citrate synthase [CS], and Complex I) samples were powdered (Mikro-Dismembrator S; Satorius®, Göttingen, Deutschland), and subsequently homogenized in ice-cold Chappel-Perry medium (0.1 M of KCl, 0.05 M of Tris, 0.01 M of MgCl_2_-6H_2_O, 1 mM of EGTA, pH 7.5) (45). Muscle enzyme activity was measured via photometry (Libra; Biochrom Ltd.®, Cambridge, England). Analysis of LDH activity was performed as described previously (45). Faloona and Srere's approach (46) was applied for measurement of CS and Hatefi and Stiggall's method (47) for Complex I. A BCA Protein Assay Kit (Pierce, Rockford, IL, USA) and a multilabel reader (PerkinElmer Precisely Victor X4 ver. 4.0; PerkinElmer Life and Analytical Science, Turku, Finland) were used to calculate protein content in muscle fibers. Muscle enzyme activity was determined in relation to protein content.

In serum, analysis of CK activity was conducted at the Department of Clinical Chemistry, University of Goettingen using automated chemistry analyzer Architect c16000 (Abbott®, Wiesbaden, Deutschland) and commercially available kits (Abbott) according to the manufacturer's instructions (Abbott). The method with N-acetyl-L-cysteine as the enzyme reactivator was applied (7D63-30, Abbott).

### Analysis of Intramuscular Fat Content

According to the International Organization for Standardization method 1443:1973, 5–10 g of the homogenized quadriceps femoris muscle were boiled in hydrochloric acid (HCl) (4 M) for 1.5 h to free the occluded and bound lipid fractions. After filtration of the resulting mass and drying, the content of the filter was extracted with light petroleum for 4 h using a Soxhlet apparatus (LAT GmbH, Garbsen, Germany). The fat content was determined as percentage of wet weight of sample taken for the analysis.

### Statistical Analysis

Statistical analyses were performed with GraphPad Prism ver. 8.2.1 (GraphPad Software, San Diego, CA, USA). For parametric data, a one-way analysis of variation (ANOVA) was used to reveal the impact of the treatments. The differences among the groups were analyzed by applying Tukey's *post-hoc* test. For non-parametric data, the Kruskal-Wallis test and the Dunn test were applied. The *p-*values less than 0.05 were considered significant. Data are presented as means and standard deviations.

## Results

### Experiment I: Ostarine

#### OS Dosage and Food Intake

**Figure 2:**
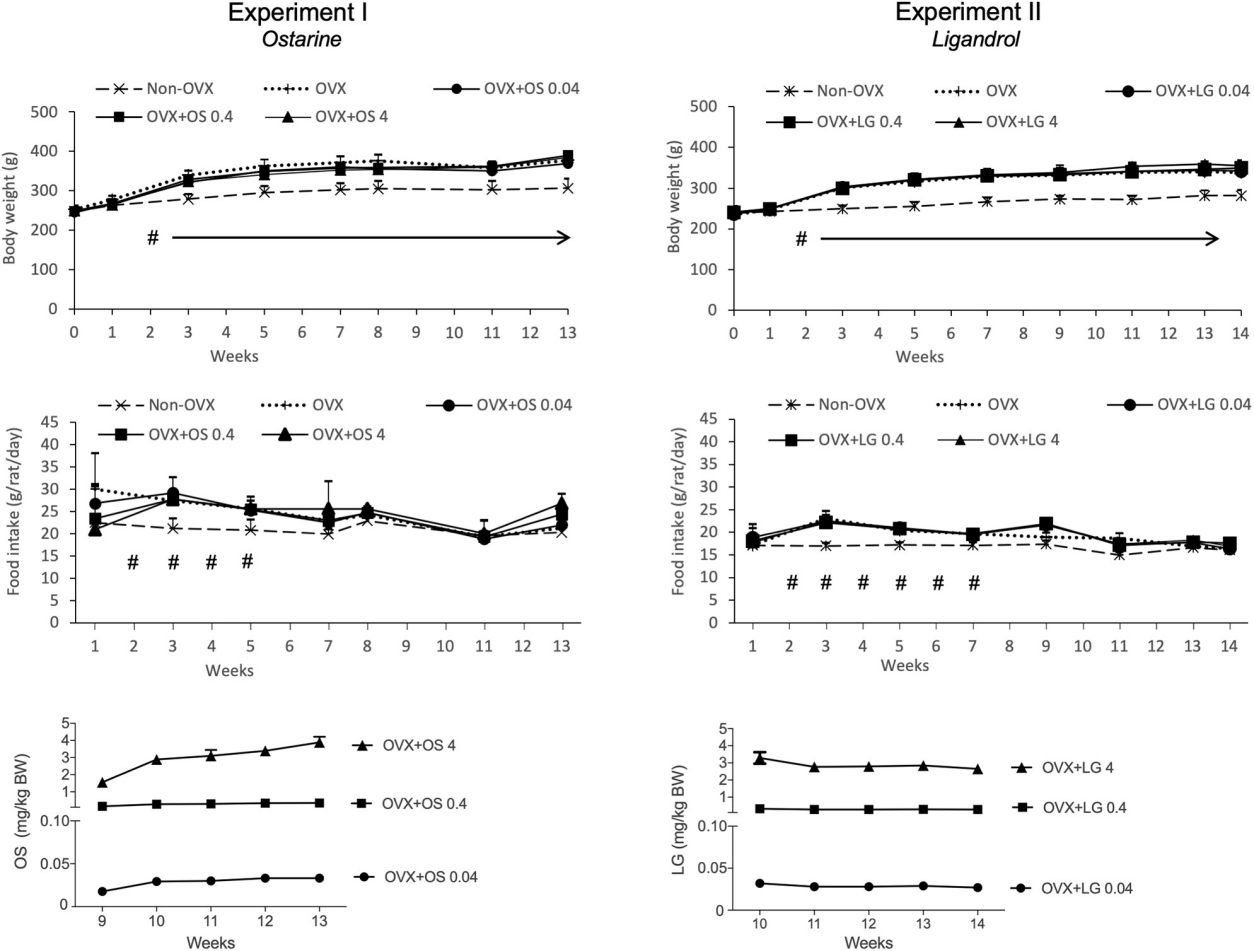
Body weight (g), daily food intake (g/rat/day) and daily intake of OS or LG (mg/kg BW) in both experiments. Data of Experiment I has been partly published (32, 33). ^#^*p* < 0.05 Non-OVX vs. all the other groups, arrows: BW in Non-OVX group differs significantly from the other groups from week 2 until the end of the study (*p* < 0.05, Tukey-Test).

The average daily uptake of OS calculated based on the food intake and BW on the respective week was 0.03 ± 0.006 mg/kg/BW in the OVX+OS 0.04 group, 0.3 ± 0.07 mg/kg/BW in the OVX+OS 0.4 group, and 3.0 ± 0.83 mg/kg/BW in the OVX+OS 4 group. The average daily food intake was 22.3 ± 4.6 g/rat/day in all groups (33). Rats in the Non-OVX group consumed less food during weeks 2 to 5 than rats in all the other groups (Figure 2).

#### Weight of the Body, Uterus, and Muscle

The BW of the rats across all treatment groups was similar at the beginning of the study (244 ± 7.8 g) (32, 33). Ovariectomy led to a significant enhancement of BW in all four OVX groups compared with the Non-OVX group from week 2 post ovariectomy. Treatment with OS did not have any influence on body weight (Table 1, Figure 2) (32, 33).

**Table 1:** Data of Experiment I: Ostarine (OS).

**Groups**	**Non-OVX**		**OVX**			**OVX+OS 0.04**			**OVX+OS 0.4**			**OVX+OS 4**			**ANOVA *p*-value**
**Sample size**	**12**		**12**			**13**			**13**			**13**			
**Parameters**	**Mean**	**SD**	**Mean**		**SD**	**Mean**		**SD**	**Mean**		**SD**	**Mean**		**SD**	
**Weights** (32, 33)															
Body weight (begin of trial) [g]	247.42	6.09	244.33		9.77	241.08		8.98	244.54		6.55	241.15		6.08	0.210
Body weight (begin of treatment) [g]	305.40	21.69	375.60	^a^	12.27	356.11	^a^	18.00	360.50	^a^	21.13	353.00	^a^	28.62	<0.001
Body weight (end of trial) [g]	307.20	29.12	376.56	^a^	19.18	368.91	^a^	23.91	389.70	^a^	32.44	385.30	^a^	25.14	<0.001
Uterus weight [g]	0.52	0.10	0.10	^a^	0.04	0.11	^a^	0.03	0.33	^abc^	0.06	0.40	^abc^	0.06	<0.001
GM weight [g]	1.87	0.27	2.02		0.26	2.21	^a^	0.29	2.26	^a^	0.26	2.27	^a^	0.24	0.005
GM weight/BW [mg/g]	6.08	0.88	5.38		0.69	6.00		0.78	5.79		0.66	5.88		0.63	0.280
SM weight [g]	0.15	0.03	0.15		0.02	0.16		0.02	0.18		0.03	0.17		0.02	0.047
SM weight/BW [mg/g]	0.48	0.09	0.41		0.05	0.44		0.05	0.45		0.08	0.44		0.05	0.246
**Muscle fibers**															
GM															
STO+FTO															
Area (μm^2^)	1863.17	400.70	2222.06		483.36	1985.55		240.70	2269.35		371.32	2248.09		551.21	0.125
Diameter (μm)	48.04	5.30	52.42		6.00	49.78		3.02	53.07		4.28	52.74		6.00	0.117
FTG															
Area (μm^2^)	4587.12	374.38	4691.65		735.21	5025.66		672.28	5463.72		1284.49	5397.56		1146.98	0.124
Diameter (μm)	75.82	3.18	76.43		6.34	79.27		5.31	82.34		8.77	81.93		8.65	0.117
LM															
STO+FTO															
Area (μm^2^)	1904.72	404.03	2226.74		437.36	2385.09		725.93	2476.78		608.82	2469.54		345.07	0.098
Diameter (μm)	48.57	5.11	52.44		5.22	53.95		7.90	55.08		6.94	55.38		3.87	0.087
FTG															
Area (μm^2^)	4634.02	734.27	5258.92		858.52	5624.63		1518.75	5830.73		1065.73	5731.07		768.68	0.082
Diameter (μm)	76.19	6.25	81.21		6.44	83.43		11.10	85.45		7.97	84.79		5.70	0.072
SM															
STO															
Area (μm^2^)	3665.93	501.22	3568.44		506.04	3973.02		478.48	3609.49		438.43	4175.71		778.99	0.072
Diameter (μm)	67.57	4.66	66.74		4.85	70.44		4.37	67.23		4.01	72.05		6.66	0.084
Percentage of fibers (%)															
STO+FTO	42.5	10.3	47.7		8.5	43.8		9.8	45.1		3.6	46.4		3.7	0.689
FTG	57.5	10.3	52.3		8.5	56.2		9.8	54.9		3.6	53.6		3.7	0.689
**Creatine kinase**															
Serum CK level (U/l)	5775.90	2001.34	6096.67		1960.97	9492.10		1691.25	6018.50		1599.74	6145.90		1456.36	0.927

*Tukey test*.

a *p < 0.05 vs. Non-OVX*.

b *p < 0.05 vs. OVX*.

c *p < 0.05 vs. OVX+OS 0.04*.

*SD, Standard deviation*.

In all four OVX groups, uterine weight was significantly lower compared with the Non-OVX group (Non-OVX group: 0.52 ± 0.10 g; OVX group: 0.10 ± 0.04 g) (32, 33). In the OVX+OS 0.04 group, uterine weight did not differ from that in the OVX group, whereas in groups OVX+OS 0.4 and OVX+OS 4 it was significantly higher than in the OVX and OVX+OS 0.04 groups (Table 1) (32, 33).

The treatments with OS resulted in a significantly higher weight of the GM than in the Non-OVX, irrespective of dosages applied. The weight of the SM did not differ between the groups. After correction by BW, the effects were not significant (Table 1) (32).

#### Vascularization of Muscles

**Figure 3:**
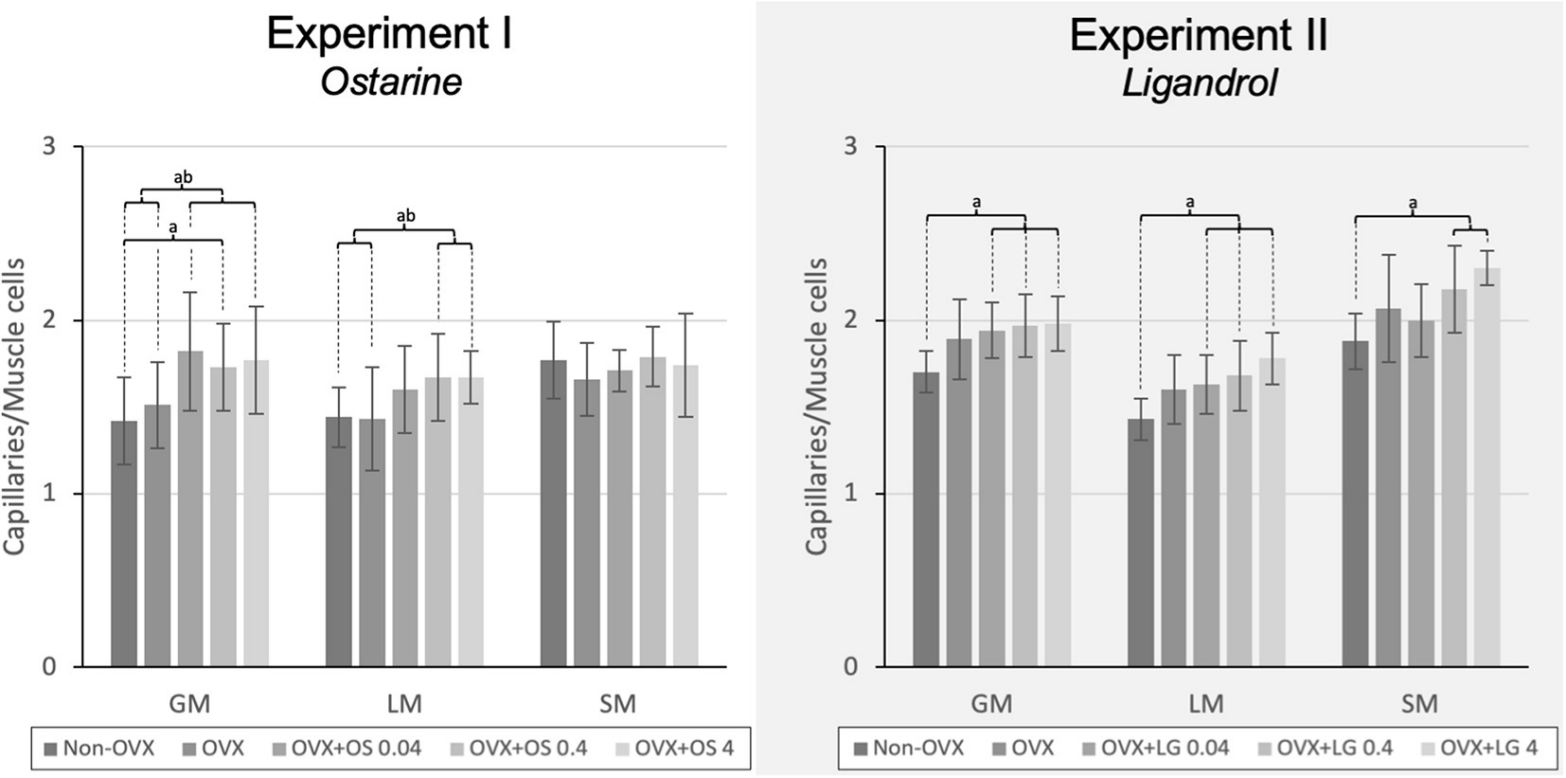
The capillary density in the LM, GM, and SM in Experiment I: Ostarine (OS) and Experiment II: Ligandrol (LG). Tukey test: a: *p* < 0.05 vs. Non-OVX, b: *p* < 0.05 vs. OVX, c: *p* < 0.05 vs. OVX+OS 0.04, d: *p* < 0.05 vs. OVX+OS 0.4.

The treatment with OS resulted in a higher capillary density in GM and LM. In the GM, all OS treatments showed a higher capillary density compared with that in the Non-OVX group. In the OVX+OS 0.04 and OVX+OS 4 groups, a significantly higher capillary density was found than in the OVX group. In the LM, a significantly higher capillary density was observed in the OVX+OS 0.4 and OVX+OS 4 treatment groups than in the Non-OVX and OVX groups. In the SM, OS treatment did not change the number of capillaries (Figure 3).

#### Muscle Fiber Size and Distribution

The treatments with OS did not result in significant alterations of the area or the corresponding diameter of STO/FTO and FTG fibers of GM, LM, or SM compared with the Non-OVX and the OVX groups. This was independent of the OS dosage. Regarding the distribution of muscle fibers in LM (FTG and STO/FTO), there were no differences (Table 1).

#### Activity of Enzymes in Muscle and Serum

**Figure 4:**
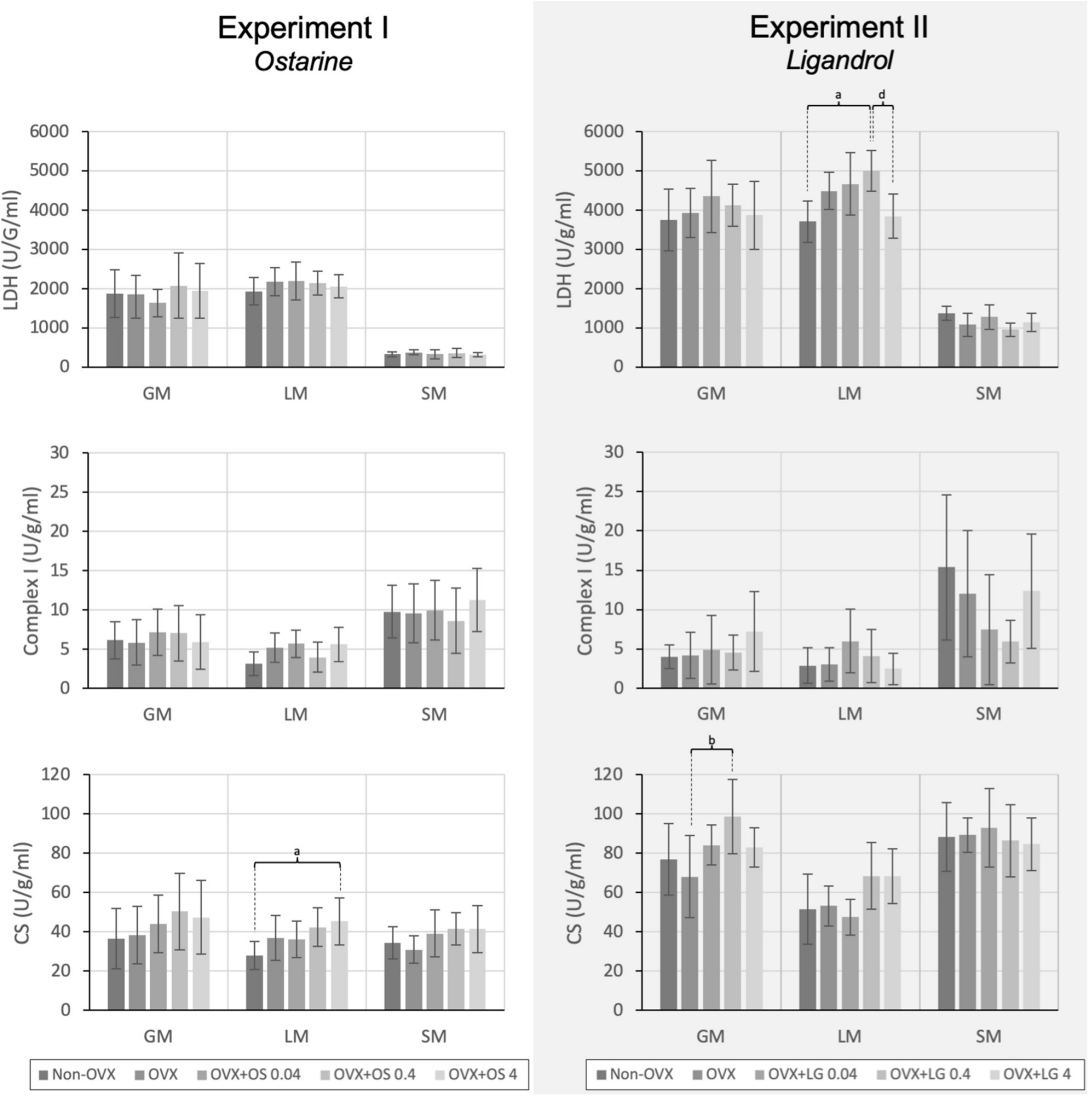
The activity of the LDH, Complex I, and CS in the GM, LM, and SM for Experiment I: Ostarine (OS) and Experiment II: Ligandrol (LG). Tukey test: a: *p* < 0.05 vs. Non-OVX, b: *p* < 0.05 vs. OVX, c: *p* < 0.05 vs. OVX+OS 0.04, d: *p* < 0.05 vs. OVX+OS 0.4.

The treatment with OS resulted in a significantly higher activity of CS in the LM in the OVX+OS 4 treatment group than in the Non-OVX group (Figure 4). This effect was not observed in the GM or the SM. LDH and Complex I activities did not differ among the groups in all three muscles studied. Serum CK levels were not changed following OS treatments after OVX (Table 1).

### Experiment II: LG

#### LG Dosage and Food Intake

The daily dosage of LG was calculated based on the food intake and BW at the respective week and averaged: 0.03 ± 0.002 mg/kg/BW in the OVX+LG 0.04 group, 0.3 ± 0.03 mg/kg/BW in the OVX+LG 0.4 group, and 3.0 ± 0.27 mg/kg/BW in the OVX+LG 4 group. The average daily food intake was 19.0 ± 2.1 g/rat/day in all groups. The food intake of Non-OVX rats was lower than in all the other groups on weeks 2 to 7 (Figure 2).

#### Weight of the Body, Uterus, and Muscle

At the beginning of the study, the rats of all treatment groups had a similar BW (237 ± 11.7 g). From week 2, the Non-OVX animals weighed significantly less than all other rats that underwent the OVX operation. This difference remained until the end of the study, irrespective of LG treatments (Table 2, Figure 2).

**Table 2:** Data of Experiment II: Ligandrol (LG).

**Groups**	**Non-OVX**		**OVX**			**OVX+LG 0.04**			**OVX+LG 0.4**			**OVX+LG 4**			**ANOVA p-value**
**Sample Size**	**12**		**12**			**13**			**13**			**13**			
**Parameters**	**Mean**	**SD**	**Mean**		**SD**	**Mean**		**SD**	**Mean**		**SD**	**Mean**		**SD**	
**Weights**															
Body weight (begin of trial) [g]	239.20	10.92	235.00		10.69	237.00		12.17	240.50		14.53	236.64		10.54	0.742
Body weight (begin of treatment) [g]	273.00	11.90	332.27	^a^	17.21	335.40	^a^	19.81	333.43	^a^	25.40	337.57	^a^	31.44	<0.001
Body weight (end of trial) [g]	282.47	14.81	338.67	^a^	20.53	342.13	^a^	27.41	350.39	^a^	16.33	355.21	^a^	19.37	<0.001
Uterus weight [g]	0.58	0.11	0.11	^a^	0.02	0.11	^a^	0.02	0.13	^a^	0.02	0.43	^abcd^	0.04	<0.001
GM weight [g]	2.03	0.14	2.29	^a^	0.14	2.30	^a^	0.23	2.45	^a^	0.20	2.49	^abc^	0.17	<0.001
GM weight/BW [mg/g]	7.20	0.51	6.71		0.41	6.69		0.68	7.06		0.57	6.94		0.47	0.059
SM weight [g]	0.11	0.02	0.12		0.02	0.12		0.03	0.13		0.02	0.11		0.03	0.619
SM weight/BW [mg/g]	0.41	0.07	0.32		0.11	0.34		0.08	0.36		0.07	0.31		0.07	0.016
**Muscle fibers**															
GM															
STO+FTO															
Area (μm^2^)	2810.58	722.26	2781.12		432.03	2631.86		440.71	2888.26		517.74	2895.14		496.48	0.812
Diameter (μm)	58.75	7.28	58.69		4.43	57.09		4.55	60.23		5.50	58.72		5.44	0.734
FTG															
Area (μm^2^)	5149.24	1457.48	5263.84		897.71	5128.98		975.04	5587.79		958.43	5950.49		951.88	0.296
Diameter (μm)	79.64	10.95	81.03		6.97	79.73		7.71	83.54		7.01	85.95		6.70	0.263
LM															
STO+FTO															
Area (μm^2^)	1921.24	381.27	2154.24		507.73	2240.71		428.29	2282.34		289.65	2561.80^a^		589.89	0.018
Diameter (μm)	48.77	4.68	51.37		6.18	52.48		5.24	53.16		3.45	55.98^a^		6.35	0.024
FTG															
Area (μm^2^)	4021.84	624.27	4574.66		833.59	4872.13		981.56	4647.10		617.80	5426.81	^a^	1080.47	0.465
Diameter (μm)	70.98	5.40	75.50		7.05	77.79		7.77	76.24		5.24	82.06	^a^	7.77	0.004
SM															
STO															
Area (μm^2^)	3637.88	396.82	4186.24	^a^	470.72	3897.86		293.80	4277.79	^a^	435.12	4106.85		635.44	0.005
Diameter (μm)	66.13	6.24	72.38	^a^	4.24	69.87		2.63	73.15	^a^	3.66	71.53	^a^	5.32	0.002
Percentage of fibers (%)															
STO+FTO	46.9	4.6	43.8		4.4	43.3		4.1	43.9		5.2	45.4		5.3	0.277
FTG	53.1	4.6	56.2		4.4	56.7		4.1	56.1		5.2	54.6		5.3	0.277
**Creatine kinase**															
Serum CK level (U/l)	5843.60	1520.58	7545.07		3510.27	5601.20		1834.22	5202.38		1960.71	5598.29		1929.49	0.061
**Percentage of intramuscular fat (median [q1, q3])**															
Quadriceps femoris muscle	1.66 (1.58, 1.88)		1.78 (1.64, 1.90)									1.94 (1.82, 2.24) ^a^			0.026

*Tukey test*.

a *p < 0.05 vs. Non-/OVX*.

b *p < 0.05 vs. OVX*.

c *p < 0.05 vs. OVX+OS 0.04*.

d *p < 0.05 vs. OVX+OS 0.4*.

*SD, Standard deviation*.

*The percentage of intramuscular fat was non-parametric*.

*q1: the first quartile, which separates the lowest 25% of the data from the highest 75%*.

*q3: the third quartile, which separates the highest 25% of the data from the lowest 75%*.

A significantly lower uterine weight was observed in all four OVX groups compared with the Non-OVX group (Non-OVX group: 0.58 ± 0.11 g; OVX group: 0.11 ± 0.02 g). LG treatments in the OVX+LG 0.04 and OVX+LG 0.4 groups did not change the uterine weight. In the OVX+LG 4 group, the weight was significantly higher compared with the OVX group and the OVX+LG 0.04 and OVX+LG 0.4 treatment groups (Table 2).

All four OVX groups showed a significantly higher GM weight than in the Non-OVX group. Treatment with a high dose of LG resulted in a significantly higher GM weight than in the OVX and OVX+LG 0.04 groups. The weight of the SM did not differ among the groups. After correction by BW, there were no significant effects (Table 2).

#### Vascularization of Muscles

The treatments with LG resulted in a higher number of capillaries in all muscles studied. In the GM and the LM, all LG treated groups showed a significantly higher capillary density than in the Non-OVX group. In the SM, this effect was observed in the OVX+LG 0.4 and OVX+LG 4 groups (Figure 3).

#### Muscle Fiber Size and Distribution

In the LM, the area and the corresponding diameter of STO/FTO and FTG fibers were significantly larger in the OVX+LG 4 group compared with the Non-OVX group. In the SM, a significantly larger area and corresponding diameter of STO/FTO fibers were observed in the OVX and OVX+LG 0.4 groups than in the Non-OVX group. Additionally, the diameter of these fibers in the SM was significantly larger in OVX+LG 4 group than in the Non-OVX group. In the GM, no differences were observed. The distribution of muscle fibers (FTG and STO/FTO) in LM did not differ among the groups (Table 2).

#### Activity of Enzymes in Muscle and Serum

In the GM, a significantly higher activity of CS was observed in the OVX+LG 0.4 treatment group than in the OVX group. The activity of other muscle enzymes in the GM did not differ among the treatment groups. In the LM, the treatment with LG showed significantly higher LDH activity in the OVX+LG 0.4 group than in the Non-OVX and the OVX+LG 4 groups. In the SM, there were no differences among the treatment groups in all three enzymes studied (Figure 4). The treatment with LG or OVX did not change the serum CK levels (Table 2).

#### Intramuscular Fat

A significantly higher intramuscular fat content of the quadriceps femoris muscle was observed in the OVX+LG 4 group compared with the Non-OVX group. In OVX rats, the fat content of the muscle did not differ from that in the other groups (Table 2).

## Discussion

This study determined the effects of the treatment with the OS and LG on the muscle tissue and metabolism of ovariectomized rats as a standard model for postmenopausal osteoporosis. Both SARMs resulted in a higher muscles' vascularization in terms of a higher capillary density and elevated muscle enzymes' activity, having no hypertrophic effect on muscle fibers.

In both experiments, BW increased significantly after OVX (32, 33), that is a known response to estrogen deprivation in rats (32, 33, 48, 49) and can be explained by enhanced food intake and other metabolic changes in these rats (50). None of the treatments with the SARMs altered the BW and food intake, and that is in line with previous reports (32, 51). In contrast, Kearbey, Gao (52) observed a higher BW in OVX rats after administration of the SARM S-4 than in OVX rats. It is possible that the changes in BW depend on the specific SARM being studied (32).

Similar to the BW, the weight of the GM was higher in all OVX rats than in Non-OVX in both experiments. This appeared to be a consequence of the estrogen deprivation (48, 49, 53). Only the OVX+LG 4 treatment group showed a higher GM's weight than in the OVX and OVX+LG 0.4 groups, which is in line with the observation of increased intramuscular fat in the OVX+LG 4 group. OS has been shown to increase lean body mass and decrease total fat mass (24). However, as a higher intramuscular fat content after treatment with LG has not been reported to the best of our knowledge so far, this impact of a SARM application has to be considered as a possible side effect. Since the analysis of intramuscular fat content is very complex and was not the main focus of the study, only the groups Non-OVX, OVX and OVX+LG 4 were chosen. Albeit, only the highest dose of LG was tested, it seems to be important finding and should be investigated in the future studies.

Regarding muscle fibers, OS and LG did not change their size in the OVX rats. In the LG experiment, an enhanced fiber size was measured in the OVX, the OVX+LG 0.4, and the OVX+LG 4 groups in the SM and in the OVX+LG 4 group in the LM compared with the Non-OVX group. Increased size of muscle fibers after OVX has been observed in previous studies (53–55), whereas estrogen supplementation has been shown to have an inhibitory effect on muscle fiber diameter (55). Vajda, Hogue (51) found that, after administration of the SARM LGD-3303 in orchiectomized rats for 12 weeks, the weight of the levator ani muscle was higher than in the castrated control. Regarding the effect of testosterone, Neto, De (56) observed a larger cross-sectional area of the plantaris muscle after 15 weeks of administration than in untreated 24-month old rats. In humans, long-term testosterone administration was shown to result in a larger muscle fiber Type I area of the vastus lateralis (57). A possible reason for the absence of stronger effects on muscle fiber size in the present study could be the relative short-term administration of OS and LG.

In this study, both OS and LG showed a beneficial effect on the muscle vascularization. It is known that enhanced vascularization improves the restoration of muscle contractility (58). While an effect of increased muscle vascularization after treatment with SARMs has not yet been shown, for the administration of testosterone, a higher capillary density was observed (56, 57). In the present study, LG resulted in a higher vascularization in all three muscles studied, than in the Non-OVX group, whereas OS influenced the capillary density in the GM and LM compared with the Non-OVX and OVX groups. Thus, OS possessed a stronger and more specific impact on muscle vascularization than LG.

Activity of several muscle enzymes was determined. CS is the pace-making enzyme of the citric acid cycle that is considered as the central metabolic pathway under aerobic conditions (59). It is a marker for the mitochondrial content and the oxidative capacity of muscles and is increased by exercise training (60–62). LDH catalyzes the conversion of lactate to pyruvate and back through the oxidation process (NAD+ ⇄ NADH). It regulates the glycolysis and is therefore essential for the cell metabolism of all nearby tissues under anaerobic conditions (63). Complex I is the first enzyme in the respiratory chain in the mitochondrial membrane and essential for the normal cell functioning. This study showed that the intermediate dosage of LG resulted in a higher activity of CS than in the OVX group in the GM and a higher activity of LDH than in the Non-OVX and OVX+LG 4 in the LM. OS had less effect on muscle enzyme activity, showing a higher CS activity in OVX+OS 4 group than in the OVX group in the LM. Whereas, an elevated CS activity is a marker for an increased aerobic capacity, an enhanced activity of both enzymes, LDH and CS, seems to be necessary for a rapid muscle recovery (58, 62). Elevation of these enzyme activities following administration of SARMs might indicate an improved muscle function. Additionally, the present study revealed that serum CK as an indicator of muscle damage was not affected by either OS or LG.

Both SARMs had an uterotrophic effect at higher dosages, which indicates a reduced selectivity with increasing the dosage. The reason for the reduced tissue selectivity of SARMs caused by different scaffoldings interacting with the N-/C-terminal domains of the androgen receptor (64). The effect on the uterus was stronger for OS than for LG, since even the intermediate dosage of OS led to a significantly higher uterine weight. A similar effect on uterine weight after administration of OS has been reported previously and represents a limitation of its application at higher doses (32, 65). Investigation at the cellular level showed that OS leads to an increased percentage of Ki67-positive cells in the uterine stroma of mice and a higher proliferation of epithelial cells (66). In the present study a distinct dose-dependent uterotrophic effect was observed that is in line with previous studies (65, 66). Correspondently to the effect in uterus, capillary density in muscle responded to the SARM treatments dose-dependently. However, the effect of different doses on other muscle parameters including metabolic enzymes was not clearly distinguished.

One of the limitations of the present study is that both SARMs were applied via food and the individual dosis could vary between the animals. On the other hand, the advantage of SARMs is their oral availability that allows easy and non-invasive administration of these substances. A further limitation is, that the experimental design lacks the effect of mechanical stimulation of muscle though the positive effect of anabolic substances on muscle size and strength as well as fat-free mass is augmented by concomitant exercise (15, 28). In the future studies on SARMs this aspect could be investigated.

In conclusion, the results of the present study showed beneficial effects of both SARMs on the muscle structure and metabolism in ovariectomized rats. Both OS and LG improved muscle vascularization, and OS had a stronger effect than LG on vascularization. In contrast, LG possessed more effect on muscle metabolic enzymes by elevating LDH and CS activities, whereas OS solely resulted in a high CS activity. A hypertrophic effect on muscle fiber size was not observed under either SARM treatment. However, higher muscle weight along with enhanced intramuscular fat content after LG treatment at high doses could be considered as an unfavorable side effect, and therefore more attention needs to be paid to this aspect in further studies on SARMs. Furthermore, the uterotrophic effect of both of the studied SARMs at higher dosages might be a limitation for their application.

## Data Availability Statement

The raw data supporting the conclusions of this article will be made available by the authors, without undue reservation.

## Ethics Statement

The animal study was reviewed and approved by Ethikkommission der Universitätsmedizin Göttingen, “Von-Siebold-Str.3”, 37075 Göttingen.

## Author Contributions

Study conception, design, and financial support: SS and MK. Acquisition of data: DH and CK. Statistical analysis: DH, CK, and PR. Analysis and interpretation of data: PR, MK, and CK. Drafting of manuscript: PR and MK. Critical revision: SS, WL, and MK. All authors read and approved the final version of this manuscript.

## Conflict of Interest

The authors declare that the research was conducted in the absence of any commercial or financial relationships that could be construed as a potential conflict of interest.

## Abbreviations

ATPase: Adenosine-triphosphatase
CS: Citrate synthase
CK: Creatine kinase
FTG: Fast-twitch glycolytic (fiber type IIb)
FTO: Fast-twitch oxidative (fiber type IIa)
GM: Gastrocnemius muscle
LDH: Lactate dehydrogenase
LG: Ligandrol
LM: Longissmus muscle
Non-OVX: Non-ovariectomized (intact)
OS: Ostarine
OVX: Ovariectomized
OVX+LG: Treatment groups with Ligandrol
OVX+OS: Treatment groups with Ostarine
PAS reaction: Periodic acid-Schiff reaction
SARMs: Selective androgen receptor modulators
SM: Soleus muscle
STO: Slow-twitch oxidative (fiber type I).
